# An *In Vitro* Protocol for Propagating *Castanea sativa* Italian Cultivars

**DOI:** 10.3390/plants11233308

**Published:** 2022-11-30

**Authors:** Vera Pavese, Paola Ruffa, Silvia Abbà, Rita Lourenço Costa, Elena Corredoira, Cristian Silvestri, Daniela Torello Marinoni, Roberto Botta

**Affiliations:** 1 Dipartimento di Scienze Agrarie, Forestali e Alimentari-DISAFA, Università degli Studi di Torino, Largo Paolo Braccini 2, Grugliasco, 10095 Torino, Italy; 2 Instituto Nacional de Investigação Agrária e Veterinária I.P., Avenida da República, Quinta do Marquês, 2780-159 Oeiras, Portugal; 3 Misión Biológica de Galicia, Sede de Santiago de Compostela, Consejo Superior de Investigaciones Científicas, Avd. Vigo s/n, 15705 Santiago de Compostela, Spain; 4 Dipartimento di Scienze Agrarie e Forestali (DAFNE), Università della Tuscia, Via San Camillo De Lellis, 01100 Viterbo, Italy

**Keywords:** axillary budding, chestnut, *in vitro* culture, micropropagation

## Abstract

*Castanea sativa* cv. ‘Garrone Rosso’ and ‘Marrone di Castel del Rio’ are two of the most prized varieties in Italy due to their valuable and healthy nuts used for fresh consumption and in the confectionery industry. Despite the growing demand for chestnuts, there are constraints regarding plant propagation that hamper the renewal and new planting of orchards in different areas. *Castanea sativa* is susceptible to diseases that have caused a reduction in its area of production. For this reason, *in vitro* culture represents a valuable technique for germplasm preservation and plant multiplication enabling production of a high number of plants for use in breeding programs. Here we present an *in vitro* micropropagation protocol for Italian *Castanea sativa* cv. ‘Marrone di Castel del Rio’ and cv. ‘Garrone Rosso’ to contribute to the preservation and enhancement of the Italian germplasm. Nodal explants were used as the starting material for *in vitro* establishment. The cv. ‘Marrone di Castel del Rio’ showed a high percentage of survival explants (92%) when subjected to long bleach exposure (25 min), in contrast to what was observed for the ‘Garrone Rosso’ cultivar. Ascorbic acid was found to be the best compound to counteract phenol exudation. The MS3B and DKW media supplied with 0.5 mg/L BAP were effective for *in vitro* establishment, while the DKW medium (0.1 mg/L BAP and 0.05 mg/L IBA) was preferable for the proliferation phase. A double-layer rooting methodology was used and 35% rooting was observed with 25 mg/L IBA rooting treatment.

## 1. Introduction

The European chestnut (*Castanea sativa* Mill.), also known as the sweet chestnut, is a multipurpose tree, valued worldwide for nut and timber production [1]. In addition, this species offers a wide range of by-products that contain potentially valuable bioactive compounds, with antioxidant, anticarcinogenic and cardioprotective properties [2]. In countries with a long tradition of cultivation, chestnut agroforestry systems provide specific ecosystem services, such as the provision of food for humans and wildlife, temperature and humidity moderation, and more generally contribute to climate regulation by carbon storage, and to the conservation of traditional knowledge, as well as to valuing of the landscape [3]. During its expansion in Europe, large populations of the species grew which varied in their characteristics, including those relating to fruit traits and plant response to biotic and abiotic stresses, such as ink disease (*Phytophthora* spp.) [4], chestnut blight (*Cryphonectria parasitica)* [5,6], and the pest *Dryocosmus kuriphilus* [7]. In Italy, the spread of chestnut tree populations across the centuries has contributed to the evolution of a rich varietal heritage in different pedoclimatic areas. Currently, over 300 cultivars are described [8]; they represent a genetic heritage that should be safeguarded and preserved [9]. Among the major Italian cultivars, ‘Marrone’ is one of the most valuable and widely known across the world. It is distributed across most of the country under different names, including ‘Marrone di ChiusaPesio’, ‘Marrone di Val Susa’, ‘Marrone di Luserna’ (Piemonte), ‘Marrone di Castel del Rio’, ‘Marrone di Zocca’ (Emilia Romagna), ‘Marrone del Mugello’, ‘Marrone di Marradi’ (Toscana), ‘Marrone di Combai’, and ‘Marrone di San Zeno’ (Veneto). These ‘Marrone’ cultivars have common morphological and carpological traits and share the same SSR genotype [10,11]. Their nuts are valued for their excellent characteristics, such as the ease of episperm removal, and the flavored flesh, which is very suitable for candying.

Besides ‘Marrone’ cultivars, the widely cultivated germplasm includes both local endangered varieties, and cultivars extensively grown in specific areas. In the Piedmont Region, one of the main Italian regions for chestnut cultivation, a significant proportion of the local fresh market is supplied by the cv. ‘Garrone Rosso’. This cultivar is a vigorous plant that offers medium-to-large sized nuts and is adapted to live at higher elevations along the Alpine valleys in comparison with other cultivars. Its produce can be marketed under the P.G.I. (Protected Geographical Indication) label “Castagna Cuneo”.

A great deal of research on chestnuts has focused on the development of vegetative propagation systems able to meet the demand for elite genotypes that provide both high-quality timber or nuts and resistance/tolerance to major diseases. Since the chestnut is a difficult-to-root species, *in vitro* techniques are attracting increasing interest for the nursery production of chestnut plants as an alternative to traditional vegetative propagation methods [12]. The first studies on the *in vitro* culture of chestnuts were carried out in the early 1980s when researchers began to see it as a viable alternative to traditional methods of propagation [13,14]. As a result of these studies, the first *in vitro* chestnut plants were obtained and successfully acclimated in the field. Studies were carried out in France aimed at propagating specific genotypes resistant to ink disease through breeding [15,16].

Explant selection is the crucial step for *in vitro* culture initiation: shoot apices, axillary buds and nodal explants are reactive explants used for *Castanea* spp. *in vitro* establishment [1,12]. Vieitez et al. [5] described an experimental protocol developed for *in vitro* propagation using axillary explants of European chestnut and its hybrids with *C. crenata*.

Although *in vitro* culture protocols have already been developed for the genus *Castanea* [1,4,5,17,18,19,20,21], large-scale propagation of *C. sativa* is currently not yet possible since it is a genotype-dependent technique and requires specific protocols for each species and cultivar, making it impossible to adopt a universal protocol. For this reason, since *in vitro* culture protocols for Italian varieties are not available, this research was focused on developing a protocol to micropropagate *C. sativa* cv. ‘Garrone Rosso’ and ‘Marrone di Castel del Rio’ (genotype ‘Marrone’), two cultivars of great value and representative of the cultivated pool of chestnut varieties in North Italy.

## 2. Results and Discussion

### 2.1. Culture Establishment

**Figure 1:**
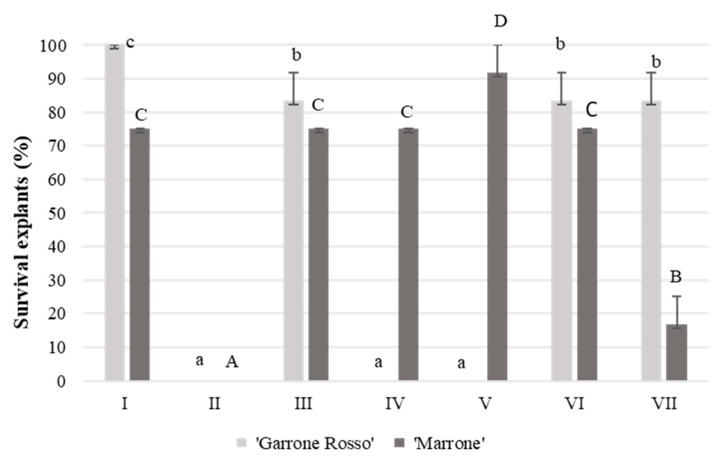
Effect of different sterilization treatments on in vitro establishment of two European chestnut cultivars ‘Marrone di Castel del Rio’ and ‘Garrone Rosso’. Treatments tested: (I) 30 s in 70% ethanol + 10 min in 33% *v*/*v* bleach + 0.2% PPM ^TM^, (II) 2% PPM ^TM^, (III) 30 s in 70% ethanol + 10 min in 33% *v*/*v* bleach, (IV) 30 s in 70% ethanol + 20 min in 33% *v*/*v* bleach + 0.2% PPM ^TM^, (V) 25 min in 33% *v*/*v* bleach, (VI) 10 min in 33% *v*/*v* bleach, (VII) 30 s in 70% ethanol + 5 min in 33% *v*/*v* bleach. Data shows the percentage of green/survival explants. In the case of treatments II, IV and V, the ‘Garrone Rosso’ cultivar showed 0% explant survival, with the same for the ‘Marrone di Castel del Rio’ cultivar in treatment II. Data are the means of three biological replicates (15 nodal explants each) ± SE. For each cultivar, means with the same letter do not significantly differ at *p* ≤ 0.05 (Tukey’s HSD test). Lowercase letters and capital letters were used for ‘Garrone Rosso’ and ‘Marrone di Castel del Rio’ respectively.

The first step of the research project involved the development of a nodal explant sterilization protocol for *C. sativa* cv. ‘Marrone di Castel del Rio’ and cv. ‘Garrone Rosso’. Sodium hypochlorite alone, or in combination with plant preservative mixture (PPM ^TM^) and ethanol 70%, was used for the sterilization trial. PPM ^TM^ is a biocide able to prevent microorganism growth [22], while ethanol 70% and sodium hypochlorite (NaOCl) are the main surface-sterilizing agents used in plant cell and tissue culture experiments [23]. Explants taken in the open field showed a high percentage level of contamination (from 80% in the case of a high exposure time to bleach to 90% for a low exposure time) in all the treatments tested. Subsequently, explants excised from plants cultivated under greenhouse conditions were used to establish an *in vitro* culture (Figure 1). The results showed differing genotype dependent behavior between the ‘Marrone di Castel del Rio’ and ‘Garrone Rosso’ cultivars. ‘Garrone Rosso’ explants, under a low exposure time to bleach (treatments I, III, VI, VII), showed a high percentage of survival explants with a statistically significant difference in treatment I (100%). In treatments II, IV and V, 0% surviving explants of ‘Garrone Rosso’ were detected; correspondingly, 100% were contaminated or necrotic. In contrast, ‘Marrone di Castel del Rio’, under a low exposure time, showed a high percentage of contamination (82% for 5 min, treatment VII). In this case, treatment V (25 min in 33% *v*/*v* of bleach) resulted in a statistically significant difference, with a high survival percentage (92%) of green healthy explants. As previously reported for other nut species [24], microbial contamination represents the main factor affecting *in vitro* culture establishment. This step is critical because it necessitates correct modulation between the concentration of the sterilizing agent and the exposure time of the explants to the solution. An establishment protocol must be appropriate, enabling removal of contamination without compromising the growth of the *in vitro* explant [5]. In conclusion, for ‘Marrone di Castel del Rio’ explants, treatment with 33% *v*/*v* bleach was successful while both ethanol and PPM ^TM^ did not improve the sterilization process; for ‘Garrone Rosso’, pretreatment in ethanol 70% for 30 s followed by 10 min in 33% *v*/*v* bleach and rinsing in PPM^TM^ was required to obtain 100% explant survival.

### 2.2. Evaluation of Polyphenol Controlling Agents to Be Applied in Chesnut In Vitro Culture

In addition to microbial contamination, another limiting factor associated with *in vitro* culture establishment of chestnut is the deleterious effect of oxidized phenolic compounds, as previously mentioned [5,12,21]. Phenolic compounds can cause apex necrosis [25] and affect plant growth.

**Figure 2:**
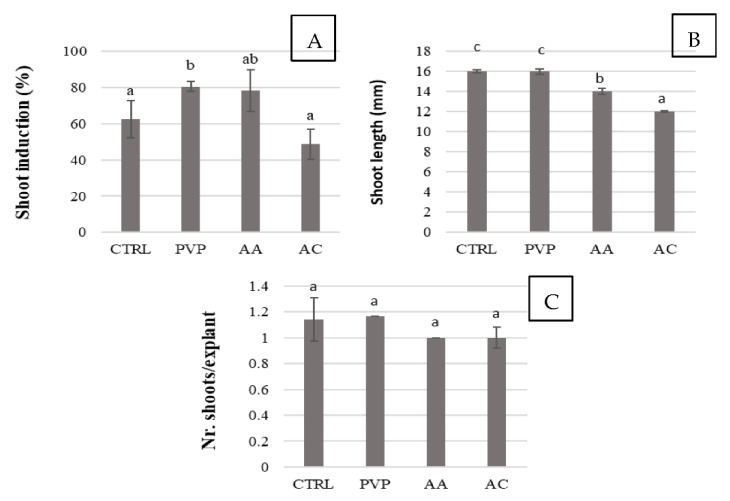
Effect of different polyphenol controlling agents on establishment of chestnut cv. ‘Garrone Rosso’. (**A**) Shoot induction percentage, (**B**) shoot length and (**C**) nr. of shoots per explant. The same results were observed for the cv. ‘Marrone di Castel del Rio’. CTR: control medium, a medium without any polyphenol controlling agents; PVP: 500 mg/L polyvinylpyrrolidone; AA: 100 mg/L ascorbic acid; AC: 1 g/L activated charcoal. Data are the means of three biological replicates (15 nodal explants each) ± SE. Means with the same letter do not significantly differ at *p* ≤ 0.05 (Tukey’s HSD test).

**Figure 3:**
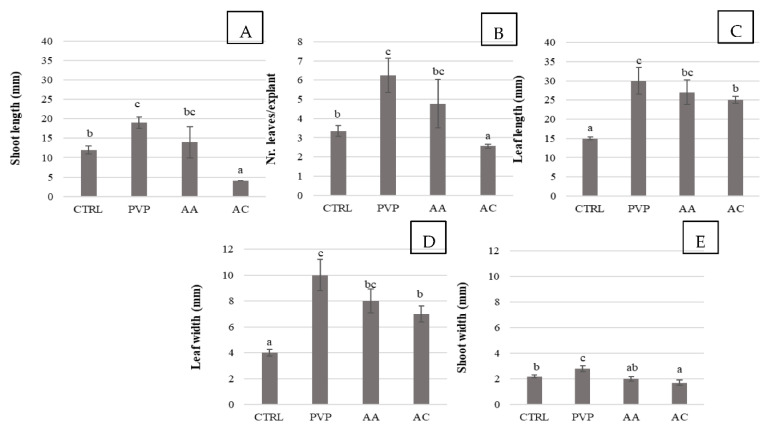
Effect of different polyphenol controlling agents on shoot proliferation of chestnut cv. ‘Garrone Rosso’. (**A**) Shoot length, (**B**) nr. of leaves per explant, (**C**) leaf length, (**D**) leaf width and (**E**) shoot width. CTR: control medium, a medium without any polyphenol controlling agents; PVP: 500 mg/L polyvinylpyrrolidone; AA: 100 mg/L ascorbic acid; AC: 1 g/L activated charcoal. Data are the means of three biological replicates (15 nodal explants each) ± SE. Means with the same letter do not significantly differ at *p* ≤ 0.05 (Tukey’s HSD test).

To counter these deleterious effects, the growth of chestnut shoots in media containing three different agents, polyvinylpyrrolidone (PVP), ascorbic acid (AA), and activated charcoal (AC) was investigated for both the establishment and proliferation steps. The results obtained revealed an important role of these molecules in promoting explant development, with PVP and AA found to be the most effective polyphenol controlling agents for *in vitro* growth in both steps (Figure 2 and Figure 3). During establishment, the addition of PVP to the culture medium resulted in the best performance in terms of the percentage of new shoots formed (80%), but not significantly different compared to the AA treatment (Figure 2). During the proliferation step, shoot length (19 mm), width (2.8 mm), number of leaves per explant, leaf length and width showed the greatest increases in the PVP treatment but were not statistically different compared to the AA treatment (Figure 3). Only shoot width was significantly larger with PVP addition compared to all other polyphenol controlling agents. Despite good development, 90% of explants exposed to PVP treatment showed evidence of necrosis on the apex, while, in the presence of AA, the plants developed without showing shoot tip necrosis. As demonstrated by North et al. [26], Nishchal et al. [27] and Ahmed et al. [28], AA and PVP act on polyphenols by reducing their negative influence and improving plant fitness. AA is an antioxidant that is also used to inhibit phenolic compound exudation and reduce oxidative browning. It does not act directly on polyphenol oxidase but prevents browning by reducing oxidized polyphenols [26]. In contrast, PVP is a water-soluble polymer that forms hydrogen bonds with phenolic compounds, promoting their precipitation [29]. In addition, it can detoxify oxygen radicals produced when the plant is injured, thus protecting cells from oxidative damage [26].

In chestnut, AA was already used in *in vitro* culture of Portuguese cultivars [1] to reduce oxidative stress, while there is no evidence of studies on the effect of PVP. Frequent cases of apex necrosis in plants grown *in vitro* are reported in the literature for different species, including chestnut [30], *Pistacia vera* [31], *Lavandula angustifolia* [32], *Quercus alba* [33], and *Juglans nigra* [34]. Our results revealed that plants cultivated on the control medium, deprived of polyphenol-controlling agents, showed polyphenol-associated stress signals, such as leaf crumpling and stem browning, which are undesirable traits in *in vitro* propagation. In contrast, explants cultivated on media with AA showed a reduction in polyphenol exudation and increased plant vitality. AA was inferred, therefore, to be the best polyphenol-controlling agent to be used in chestnut *in vitro* culture. Finally, AC was found to be of little benefit in controlling oxidized polyphenols in chestnut and caused stunted growth of shoots. The negative effect of AC contrasts with the results reported by Chevre et al. [35] who found that AC favors the elongation of *C. sativ*a cv. ‘Marigoule’ explants, compared with explants treated with PVP, which did not result in significant effects. These different results could be due to genotypic differences of the starting material [36].

**Figure 4:**
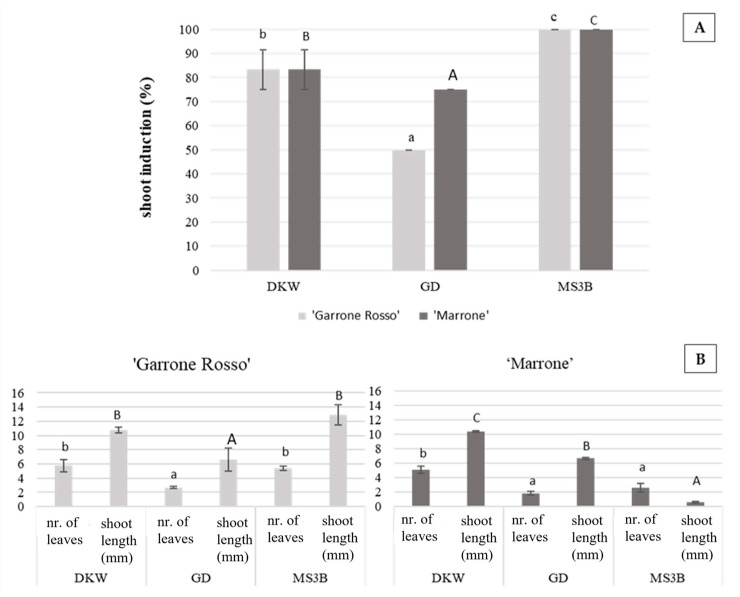
(**A**) Percentage of new induced shoots in cultivars ‘Garrone Rosso’ and ‘Marrone di Castel del Rio’. (**B**) Morphological traits of ‘Garrone Rosso’ and ‘Marrone di Castel del Rio’ in the three establishment media tested. Data are the means of three biological replicates (15 shoots each) ± SE. For each cultivar, means with the same letter do not significantly differ at *p* ≤ 0.05 (Tukey’s HSD test). Lowercase letters and capital letters were used to indicate nr. of leaves and shoot length respectively.

After detecting the best polyphenol controlling agent compound to be added in both establishment and proliferation steps, disinfected explants and bud sprouts were successfully cultured on the three-shoot induction media tested and treated with 100 mg/L AA. For both genotypes, the shoot induction percentage was significantly higher (*p* < 0.05) in the explants cultivated on Murashige and Skoog medium with half-strength NH_4_NO_3_ and KNO_3_ (MS3B) [37] compared to the GD medium [38] and to the DKW-modified medium [39,40] (Figure 4). With respect to the morphology of the shoots, in ‘Garrone Rosso’, the number of leaves was significantly higher in shoots cultivated on media containing DKW or MS3B mineral solution compared to those cultivated on the medium containing GD mineral solution. In ‘Marrone di Castel del Rio’, the number of leaves was significatively higher in plants cultivated on DKW mineral solution compared to GD and MS3B. In ‘Garrone Rosso’, the shoot length was significantly higher in plants cultivated on MS3B and DKW media than in the GD medium. In ‘Marrone di Castel del Rio’, the shoot length was significantly different between all treatments; resulting to be higher in the DKW medium.

In conclusion, both MS3B and DKW media were found to be good media for ‘Garrone Rosso’ and ‘Marrone di Castel del Rio’ shoot induction and development. To obtain a high percentage of responding explants, it is preferable to use the MS3B medium for both cultivars; in order to obtain well developed shoots, for ‘Garrone Rosso’ no difference between DKW and MS3B was detected, while, for ‘Marrone di Castel del Rio’, a significant increment in shoot size was detected in DKW medium.

### 2.3. Shoot Multiplication and Elongation

**Figure 5:**
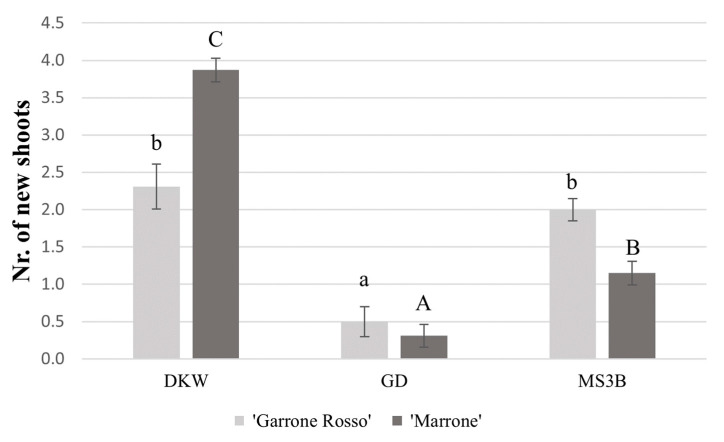
Histogram showing the multiplication rates of ‘Marrone di Castel del Rio’ and ‘Garrone Rosso’ cultivars. For each cultivar, means with the same letter do not significantly differ at *p* ≤ 0.05 (Tukey’s HSD test). Lowercase letters and capital letters were used for ‘Garrone Rosso’ and ‘Marrone di Castel del Rio’ respectively.

**Figure 6:**
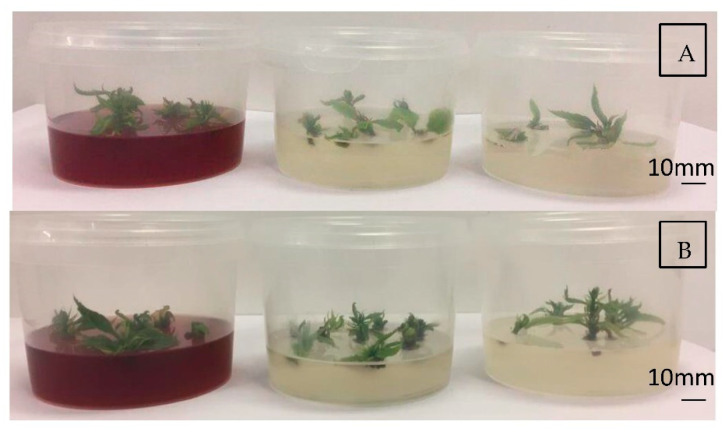
(**A**) *In vitro* shoots of ‘Marrone di Castel del Rio’ during multiplication in DKW, GD and MS3B media (from left to right). (**B**) *In vitro* shoots of ‘Garrone Rosso’ during multiplication on DKW, GD and MS3B media (from left to right).

In *Castanea* spp. micropropagation, BAP is the plant regulator most widely used, as in other woody species, such as *Quercus* spp. [41]. A high concentration of BAP (0.5–2 mg/L) is used during the establishment phase to enable the development of new shoots, then BAP, according to Fernandes et al. [1], is reduced (0.005–0.2 mg/L) in the multiplication phase. The addition of a low concentration of IBA, in the multiplication phase, facilitates shoot growth (as previously mentioned by Gürel et al. [42]; Bahri et al. [43]). The shoot multiplication rates obtained for the two different genotypes and the three media tested are shown in Figure 5. In both genotypes (Figure 6A,B), the highest multiplication rate was achieved with the modified DKW media, but with a statistically significant difference obtained only for ‘Marrone di Castel del Rio’. For ‘Marrone di Castel del Rio’ (Figure 5), the number of new shoots cultivated on DKW medium (3.87 ± 0.16) was significantly higher compared to the MS3B (1.15 ± 0.16) and GD (0.31 ± 0.15) media. For ‘Garrone Rosso’ (Figure 5), both for DKW (2.31 ± 0.3) and for MS3B (2.0 ± 0.2), the multiplication rate was significant higher compared to that for the GD medium (0.5 ± 0.15). Our results are consistent with the multiplication rates (approximately 2.5 to 3.0 of new shoots/explant) observed by Fernandes et al. [20] Ballester et al. [44] and Miranda and Fernandez [45]. Our results suggest a higher multiplication rate in DKW, which makes it a good multiplication medium for both ‘Marrone di Castel del Rio’ and ‘Garrone’ cultivars. As mentioned by Clapa et al. [46], the addition of Sequestrene 138, present in the modified DKW [40], increased the vigor of the plants as well as the length and number of new shoots.

Following Fernandes et al. [20], WPM [47] medium supplemented with zeatin was successfully used for shoot elongation of ‘Marrone di Castel del Rio’ and ‘Garrone Rosso’. For ‘Garrone Rosso’, the number of leaves and the shoot length were 7.4 ± 0.7 mm and 29.3 ± 1.96 mm, respectively, while, for ‘Marrone di Castel del Rio’, they were 10.23 ± 1.12 mm and 35 ± 2.2 mm, respectively.

### 2.4. Rooting

Rooting is the critical phase in the chestnut *in vitro* protocol.

**Figure 7:**
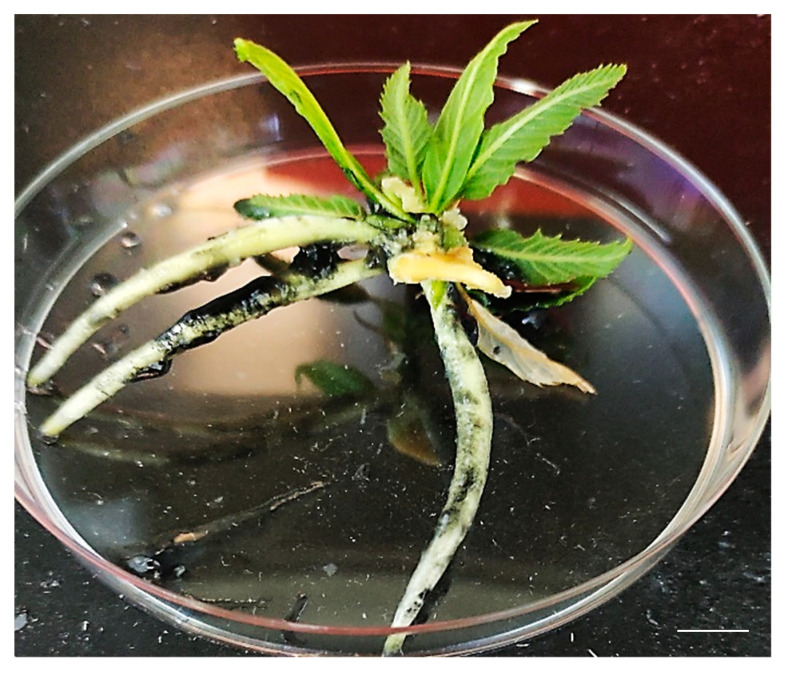
Chestnut rooting following the double-layer method. Bars = 10 mm.

Rooting capacity, in both genotypes, was significantly (Tukey’s HSD test, *p* < 0.05) affected by the rooting treatment used. The highest rooting percentage (35%) was observed when the rooting treatment was applied for three days with a high concentration (25 mg/L) of indol-3-butyric acid (IBA) (Figure 7). The length of the roots was 50 ± 1.2 mm for ‘Garrone Rosso’ and 44 ± 1.5 mm for ‘Marrone di Castel del Rio’, respectively. No rooting appearance was detected in explants subjected to a low concentration of IBA (3 mg/L) for seven days. Our results are consistent with results observed for chestnut hybrids [5] and other *Fagaceae* species, such as the holm oak [48] or American oaks [33]. The low rooting rates depend on the genotype that plays a crucial role in the micropropagation of hardwoods and affects all steps [49]. Since the high concentration of IBA was effective for rooting induction, further investigations are needed to increase the rooting percentage by testing other rooting regulators at different concentrations and for different exposure times. The double-layer method for *in vitro* rooting induction is a useful strategy to induce rooting, enabling avoidance of transferring explants to a new medium and, therefore, limiting the plant stress of subcultures.

## 3. Materials and Methods

### 3.1. Plant Material

For *in vitro* establishment, the explants were collected from both the field and the greenhouse. Scions of the cultivar ‘Garrone Rosso’ and ‘Marrone di Castel del Rio’ were grafted onto potted hybrid rootstocks (*C. sativa* x *C. crenata*) (approximately two-year-old plants) and maintained under greenhouse conditions. Grafting was undertaken in February and explants were collected in May.The first three nodal explants were used, the shoot apex discarded, and leaves removed. Then, the explants were rinsed for 1h under running tap water. In addition, young herbaceous twigs were collected under field conditions from ‘Garrone Rosso’ and ‘Marrone di Castel del Rio’ adult trees at the Centro Regionale di Castanicoltura del Piemonte, Chiusa Pesio.

### 3.2. Culture Establishment and Anti-Phenolic Treatments

For sterilization, seven different treatments were evaluated. The explants were surface sterilized in 33% *v*/*v* (4.5% of active chlorine) commercial bleach (ACE, Procter & Gamble) plus a few drops of Twin 20. The treatment with bleach was performed alone or in combination with ethanol 70% (*v*/*v*) and 2% (treatment II) or 0.2% (treatments I and IV) Plant preservative mixture (PPM^TM^) (Plant Cell Technology, Washington, DC, USA) was used as described in Table 1. After the sterilization process, the explants were rinsed twice in sterilized distilled water (10 min each). Explants were then transferred to the nutritive medium. All the media used are described in Supplementary Materials File S1.

To define the best agent to be added for polyphenol exudation control, preliminary experiments were conducted by testing the effect of 500 mg/L polyvinylpyrrolidone (PVP) [27,50], 100 mg/L ascorbic acid (AA) [1] and 1 g/L activated charcoal (AC) [26]. These polyphenol-controlling agents were added to the modified MS medium [37] containing half-strength NH_4_NO_3_ and KNO_3_. The medium included 3% sucrose and 0.5 mg/L benzylaminopurine (BAP) in the induction phase [5], and 0.1 mg/L BAP in the proliferation stage [1] plus 0.8% agar.

In a second experiment, the effect of mineral solutions on shoot establishment was evaluated. Three mineral solutions were investigated: MS medium with half-strength NH_4_NO_3_ and KNO_3_ (MS3B), the Greshoff and Doy medium (GD), and a modified DKW mineral solution, supplemented with 0.5 mg/L BAP [5], 3% sucrose and 0.8% plant agar (DUCHEFA, NL), and 100 mg/L AA (as it performed better than the others in the preliminary experiments).

In all media, the pH was adjusted to 5.6 prior to autoclaving for 20 min at 121 °C. In all experiments, explants consisting of 1–2 cm pieces, containing one single bud each, were placed in 100 × 20 mm glass tubes containing 5 mL of medium. The cultures were maintained in a growth chamber at 24 ± 1 °C with a 16 h photoperiod provided by fluorescent lamps (40 µmol m^−2^s^−1^). After three weeks in culture, the percentage survival of shoots, and the number and the length of shoots originating from a single bud, were evaluated.

### 3.3. Shoot Multiplication and Elongation

Shoots were transferred to the same mineral solutions used in the establishment step (see Section 3.1) with a reduced content of BAP (0.1 mg/L) [1] and the addition of 0.05 mg/L indole-3-butyric acid (IBA) and subcultured every six weeks. As reported by Vieitez et al. [14] and Osterc et al. [51], concentrations of BAP at 0.1–0.5 mg/L have been shown to be the most suitable for proliferation and healthy growth. At the end of the third subculture, the multiplication rates were determined by counting the number of newly developed shoots.

After the proliferation phase, the explants were transferred to the elongation media. WPM medium with 100 mg/L of AA, 30 g/L sucrose, 0.1 mg/L of zeatin and 8 g/L agar was used, following Fernandes et al. [1]. The pH was adjusted to 5.6. The elongated shoots were morphologically analyzed by measuring the shoot size and the number and length of leaves.

### 3.4. Rooting

Rooting was induced using a double-layer methodology which consisted of two media layers overlaid on each other. The lower medium layer was the elongation medium previously used in the elongation step. Two different concentrations of IBA were tested in the liquid-modified MS1B (with half-concentration of macro) upper layer: (a) 25 mg/L IBA—plants were cultured on this medium for 3 days, (b) 3 mg/L IBA—plants were cultured for 7 days [5]. After the induction of rooting, the shoots were transferred to a rooting expression medium consisting of the same mineral medium without plant growth regulators and enriched with 0.5% AC for 6 weeks.

### 3.5. Statistical Analysis

Data were subjected to analysis of variance (ANOVA) using the SPSS software for Windows (version 26.0, SPSS Inc., Chicago, IL, USA). Data recorded as percentages were transformed by arcsine square root prior to being subject to ANOVA. The data presented are the means of three technical replicates ± SE. Means with the same letter do not significantly differ at *p* ≤ 0.05 (Tukey’s HSD test).

## 4. Conclusions

A suitable protocol for the *in vitro* culture of chestnut Italian cultivars was set up. The results highlighted that the best *in vitro* compound for polyphenols control was the AA, which resulted in the production of healthy and well-developed plants. MS3B and DKW were found to be the best mineral solutions for the ‘Marrone di Castel del Rio’ and ‘Garrone Rosso’ cultivar *in vitro* establishment, while DKW resulted in a better multiplication rate in the proliferation phase. Further investigations are needed to increase the rooting percentage by testing other rooting regulators at different concentrations and for different exposure times, as rooting is the critical step for the production of woody species.

This protocol represents a useful source for use for future breeding purposes.

## Figures and Tables

**Table 1:** Treatments tested for explant sterilization.

Sterilization Treatments Tested	
I	30 s in 70% ethanol + 10 min in 33% *v*/*v* bleach + 0.2% PPM^TM^
II	2% PPM^TM^
III	30 s in 70% ethanol + 10 min in 33% *v*/*v* bleach
IV	30 s in 70% ethanol + 20 min in 33% *v*/*v* bleach + 0.2% PPM^TM^
V	25 min in 33% *v*/*v* bleach
VI	10 min in 33% *v*/*v* bleach
VII	30 s in 70% ethanol + 5 min in 33% *v*/*v* bleach

PPM: Plant preservative mixture.

## Data Availability

Not applicable.

## Supplementary Materials

The following supporting information can be downloaded at: https://www.mdpi.com/article/10.3390/plants11233308/s1, File S1: media used for chestnut *in vitro* culture.

## References

[1] Fernandes P., Tedesco S., Vieira da Silva I., Santos C., Machado H., Lourenço Costa R. (2020). A new clonal propagation protocol develops quality root systems in Chestnut. Forests.

[2] Braga N., Rodrigues F., Beatriz M., Oliveira P.P. (2015). *Castanea sativa* by-products: A review on added value and sustainable application. Nat. Prod. Res..

[3] Roces-Díaz J.V., Díaz-Varela E.R., Barrio-Anta M., Álvarez-Álvarez P. (2018). Sweet chestnut agroforestry systems in north-western Spain: Classification, spatial distribution and an ecosystem services assessment. For. Syst..

[4] Gago D., Bernal M.Á., Sánchez C., Aldrey A., Cuenca B., Christie C.B., Vidal N. (2022). Effect of sucrose on growth and stress status of *Castanea sativa* x *C. crenata* shoots cultured in liquid medium. Plants.

[5] Vieitez A.M., Sänchez M.C., García-Nimo M.L., Ballester A., Jain S.M., Haggman H. (2007). Protocol for micropropagation of *Castanea sativa*. Protocols for Micropropagation of Woody Trees and Fruits.

[6] Pavese V., Moglia A., Gonthier P., Torello Marinoni D., Cavalet-Giorsa E., Botta R. (2021). Identification of susceptibility genes in *Castanea sativa* and their transcription dynamics following pathogen infection. Plants.

[7] Sartor C., Dini F., Marinoni D.T., Mellano M.G., Beccaro G.L., Alma A., Quacchia A., Botta R. (2015). Impact of the Asian wasp *Dryocosmuskuriphilus* (Yasumatsu) on cultivated chestnut: Yield loss and cultivar susceptibility. Sci. Hortic..

[8] Beccaro G., Alma A., Bounous G., Gomes-Laranjo J., Beccaro G., Alma A., Bounous G., Gomes-Laranjo J. (2019). The Chestnut Handbook: Crop & Forest Management.

[9] Bounous G., Ertürk U., Akyuz B., Fulbright D.W., Serdar U. (2017). Evaluation of the descriptive characteristics of chestnut. VI Int. Chestnut Symp..

[10] Torello Marinoni D., Akkak A., Guaraldo P., Boccacci P., Ebone A., Viotto E., Bounous G., Ferrara A.M., Botta R. (2013). Genetic and morphological characterization of chestnut (*Castanea sativa* Mill.) germplasm in Piedmont (north-western Italy). Tree Genet. Genomes.

[11] Alessandri S., Krznar M., Ajolfi D., Ramos Cabrer A.M., Pereira-Lorenzo S., Dondini L. (2020). Genetic diversity of *Castanea sativa* Mill. accessions from the Tuscan-Emilian Apennines and Emilia Romagna Region (Italy). Agronomy.

[12] Merkle S.A., Viéitez F.J., Corredoira E., Carlson J.E., Litz R.E., Fernando Pliego-Alfaro A., Hormaza J.I. (2020). *Castanea* spp. Chestnut. Biotechnology of Fruit and Nut Crops.

[13] Vieitez A.M., Vieitez M.L. (1980). Culture of chestnut shoots from buds *in vitro*. J. Hortic. Sci..

[14] Vieitez A.M., Ballester A., Vieitez M.L., Vieitez E. (1983). *In vitro* plantlet regeneration of mature chestnut. J. Hortic. Sci..

[15] Chevre A., Salesses G. (1987). Choice of explants for chestnut micropropagation. Symposium on In Vitro Problems Related to Mass Propagation of Horticultural Plants.

[16] Chauvin J.E., Salesses G. (1988). The effect of fructose on chestnut micropropagation *Castanea* sp.. ComptesRendus De L’academieDesSci. Ser. 3 Sci. Vie.

[17] Liu Z., Bi W.-L., Shukla M.R., Saxena P.K. (2022). In vitro technologies for American chestnut (*Castanea dentata* (Marshall) Borkh) conservation. Plants.

[18] Gaidamashvili M., Khurtsidze E., Kutchava T., Lambardi M., Benelli C. (2021). Efficientprotocolforimprovingthedevelopmentofcryopreservedembryonicaxesofchestnut (*Castanea sativa* Mill.) byencapsulation-vitrification. Plants.

[19] Freitas T.R., Santos J.A., Silva A.P., Fraga H. (2021). Influence of climate change on chestnut trees: A Review. Plants.

[20] Fernandes P., Amaral A., Colavolpe B., Balonas D., Serra M., Pereira A., Costa R.L. (2020). Propagation of new chestnut rootstocks with improved resistance to *Phytophthora cinnamomi*. New Cast Rootstocks. Silva Lusit..

[21] Corredoira E., Martínez M.T., Cernadas M.J., San José M.C. (2017). Application of biotechnology in the conservation of the genus Castanea. Forest.

[22] Çölgeçen H., Çalișkan U.K., Toker G. (2011). Influence of different sterilization methods on callus initiation and production of pigmented callus in *Arnebiadensiflora*Ledeb. Turk. J. Biol..

[23] Ma M., Zhao L., Tang S., Chen X., Qin R. (2018). The Effects of Different Disinfection Methods on Seed Germination and Study on the Environmental Bacteria in Safflower (*Carthamus tinctorius* L.). Crops.

[24] Aliyu O.M., Awopetu J.A. (2005). In vitro regeneration of hybrid plantlets of cashew (*Anacardium occidentale* L.) through embryo culture. Afr. J. Biotechnol..

[25] Teixeira da Silva J.A., Nezami-Alanagh E., Barreal M.E., Kher M.M., Wicaksono A., Gulyás A., Hidvégi N., Magyar-Tábori K., Mendler-Drienyovszki N., Márton L. (2020). Shoot tip necrosis of in vitro plant cultures: A reappraisal of possible causes and solutions. Planta.

[26] North J.J., Ndakidemi P.A., Laubscher C.P. (2012). Effects of antioxidants, plant growthregulators and wounding on phenolic compound excretion during micropropagation of *Strelitzia reginae*. Int. J. Phys. Sci..

[27] Nishchal N., Mir H., Rani R., Pal A.K. (2018). Effect of antioxidants in controlling phenol exudation in micropropagation of litchi cv. Purbi. Curr. J. Appl. Sci. Technol..

[28] Ahmed A.B.A., Rao A.S., Rao M.V., Taha R.M. (2011). Effect of picloram, additivesand plant growth regulators on somatic embryogenesis of *Phyla nodiflora* (L.) Greene. Braz. Arch. Biol. Technol..

[29] Arif I.A., Bakir M.A., Khan H.A., Ahamed A., Al Farhan A.H., Al Homaidan A.A., Al Sadoon M., Bahkali A.H., Shobrak M. (2010). A simple method for DNA extraction from mature date palm leaves: Impact of sand grinding and composition of lysis buffer. Int. J. Mol. Sci..

[30] Vieitez A.M., Sánchez C., San-José C. (1989). Prevention of shoot-tip necrosis in shoot cultures of chestnut and oak. Sci. Hortic..

[31] Barghchi M., Alderson P.G. (1985). In vitro propagation of *Pistacia vera* L. and thecommercial cultivars Ohadi and Kalleghochi. J. Hortic. Sci..

[32] Machado M.P., da Silva A.L.L., Biasi L.A., Deschamps C., Filho J.C.B., Zanette F. (2014). Influence of calcium content of tissue on hyperhydricity and shoot-tip necrosis ofin vitroregenerated shoots of *Lavandula angustifolia* Mill. Braz. Arch. Biol. Technol..

[33] Vieitez A.M., Corredoira E., Ballester A., Muñoz F., Durán J., Ibarra M. (2009). *In vitro*regeneration of the important North American oak species *Quercus alba*, *Quercus bicolor* and *Quercus rubra*. Plant Cell Tissue Organ Cult..

[34] Bosela M.J., Michler C.H. (2008). Media effects on black walnut (*Juglans nigra* L.) shoot culture growth in vitro: Evaluation of multiple nutrient formulations and cytokinin types. In Vitro Cell. Dev. Biol..

[35] Marie Chevre A., Gill S.S., Mouras A., Salesses G. (1983). In vitro vegetative multiplication of chestnut. J. Hortic. Sci..

[36] Abdelwahd R., Hakam N., Labhilili M., Udupa S. (2008). Use of an adsorbent and antioxidants to reduce the effects of leached phenolics in *in vitro* plantlet regeneration of faba bean. Afr. J. Biotechnol..

[37] Murashige T., Skoog F. (1962). A revised medium for rapid growth and bio assays with tobacco tissue cultures. Physiol. Plant.

[38] Gresshoff P.M., Doy C.H. (1972). Haploid Arabidopsis thaliana callus and plants from anther culture. Aust. J. Biol. Sci..

[39] Driver J.A., Kuniyuki A.H. (1984). In vitro propagation of Paradox walnut rootstock. HortScience.

[40] Silvestri C., Rugini E., Cristofori V. (2019). The effect of CuSO4 for establishing in vitro culture, and the role nitrogen and iron sources in in vitro multiplication of *Corylus avellana* L. cv. Tonda Gentile Romana. Plant Biosyst..

[41] Vieitez A.M., Corredoira E., Martínez M.T., José M.C.S., Sánchez C., Valladares S., Vidal N., Ballester A. (2012). Application of biotechnological tools to *Quercus* improvement. Eur. J. For. Res..

[42] Gürel S., Gülșen Y. (1998). The effects of IBA and BAP on in vitro shoot production of almond (*Amygdalus communis* L.). Turk. J. Biol..

[43] Bahri N.B., Bettaieb T. (2013). In vitro propagation of a forest tree *Paulownia tomentosa* (Thunb.) Steud.-A valuable medicinal tree species. Albanian J. Agric. Sci..

[44] Ballester A., Bourrain L., Corredoira E., Gonçalves J.C., Lê C.-L., Miranda-Fontaíña E., San José M.C., Sauer U., Viéitez Martín A.M. (2001). Improving chestnut micropropagation through axillary shoot development and somatic embryogenesis. For. Snow Landsc. Res..

[45] Miranda M., Fernandez J. (2001). Genotypic and environmental variation of *Castanea crenata* x *C. sativa* and *Castanea sativa* clones in aptitude to micropropagation. Silvae Genet..

[46] Clapa D., Bunea C., Borsai O., Pintea A., Hârța M., Ştefan R., Fira A. (2018). The role of Sequestrene 138 in highbush blueberry (*Vaccinium corymbosum* L.) micropropagation. HortScience.

[47] Lloyd G., McCown B. (1980). Commercially feasible micropropagation of mountain laurel *Kalmia latifolia* by use of shoot tip culture. Comb. Proc. Int. Plant Prop. Soc..

[48] Martínez M.T., Vieitez F.J., Solla A., Tapias R., Ramírez-Martín N., Corredoira E. (2020). Vegetative propagation of *Phytophthora cinnamomi*-tolerant holm oak genotypes by axillary budding and somatic embryogenesis. Forests.

[49] Martínez M.T., Arrillaga I., Sales E., Pérez-Oliver M.A., González-Mas M.D.C., Corredoira E. (2021). Micropropagation, characterization, and conservation of *Phytophthora cinnamomi*-tolerant holm oak mature trees. Forests.

[50] Thomas T.D. (2008). The role of activated charcoal in plant tissue culture. Biotechnol. Adv..

[51] Osterc G., Fras M.Z., Vonedik T., Luthar Z. (2005). The propagation of chestnut (*Castanea sativa* Mill.) nodal explants. Acta Agric. Slov..

