# Supplementary material for: An In Vitro Protocol for Propagating Castanea sativa Italian Cultivars
Source: Plants (Basel). 2022 Nov 30;11(23):3308. doi: 10.3390/plants11233308 (PMC9738486; doi:10.3390/plants11233308)
Supplement: Supplementary file 1 [file plants-11-03308-s001.zip › plants-1990724-supplementary.pdf]

Supplementary File S1: media used for chestnut *in vitro* culture

Mineral solutions

DKW (DUCHEFA, NL)	
**Micro Elements**	**mg/l**
CuSO4.5H2O	0.25
FeNaEDTA	44.63
H3BO3	4.80
MnSO4.H2O	33.80
Na2MoO4.2H2O	0.39
ZnSO4.7H2O	17.00
**Macro Elements**	**mg/l**
CaCl2	112.50
Ca(NO3)2.2H2O	1664.64
KH2PO4	265.00
K2SO4	1559.00
MgSO4	361.49
NH4NO3	1416.00
**Vitamins**	**mg/l**
Glycine	2.00
myo-Inositol	100.00
Nicotinic acid	1.00
Thiamine HCl	2.00

MS3B (DUCHEFA, NL)	
**Micro Elements**	**mg/l**
$CoCl_2.6H_2O$	0.025
$CuSO_4.5H_2O$	0.025
FeNaEDTA	36.70
H_3BO_3	6.20
KI	0.83
$MnSO_4.H_2O$	16.90
$Na_2MoO_4.2H_2O$	0.25
$ZnSO_4.7H_2O$	8.60
**Macro Elements**	**mg/l**
$CaCl_2$	332.02
KH_2PO_4	170.00
KNO_3	950.00
$MgSO_4$	180.54
NH_4NO_3	825.00
**Vitamins**	**mg/l**
Glycine	2.00
myo-Inositol	100.00
Nicotinic acid	0.50

Pyridoxine HCl	0.50
Thiamine HCl	0.10
**GD (DUCHEFA, NL)**	
**Micro Elements**	**mg/l**
$CoCl_2.6H_2O$	0.025
$CuSO_4.5H_2O$	0.025
FeNaEDTA	36.70
H_3BO_3	0.30
KI	0.80
$MnSO_4.H_2O$	1.00
$Na_2MoO_4.2H_2O$	0.025
$ZnSO_4.7H_2O$	0.30
**Macro Elements**	**mg/l**
$Ca(NO_3)2.2H_2O$	208.81
KCl	65.00
KH_2PO_4	300.00
KNO_3	1000.00
$MgSO_4$	17.09
NH_4NO_3	1000.00
**Vitamins**	**mg/l**
Glycine	4.00
myo-Inositol	100.00
Nicotinic acid	1.00
Pyridoxine HCl	1.00
Thiamine HCl	10.00
**WPM (DUCHEFA, NL)**	
**Micro Elements**	**mg/l**
$CuSO_4.5H_2O$	0.25
FeNaEDTA	36.70
H_3BO_3	6.20
$MnSO_4.H_2O$	22.30
$Na_2MoO_4.2H_2O$	0.25
$ZnSO_4.7H_2O$	8.60
**Macro Elements**	**mg/l**
$CaCl_2$	72.50
$Ca(NO_3)2 .4H_2O$	471.26
KH_2PO_4	170.00
K_2SO_4	990.00
$MgSO_4$	180.54
NH_4NO_3	400.00
**Vitamins**	**mg/l**
Glycine	2.00
myo-Inositol	100.00

Nicotinic acid	0.50
Pyridoxine HCl	0.50
Thiamine HCl	1.00

MS1B (DUCHEFA, NL)	
**Micro Elements**	**mg/l**
$CoCl_2.6H_2O$	0.025
$CuSO_4.5H_2O$	0.025
FeNaEDTA	36.70
H_3BO_3	6.20
KI	0.83
$MnSO_4.H_2O$	16.90
$Na_2MoO_4.2H_2O$	0.25
$ZnSO_4.7H_2O$	8.60
**Macro Elements**	**mg/l**
$CaCl_2$	166.00
KH_2PO_4	85.00
KNO_3	950.00
$MgSO_4$	87.86
NH_4NO_3	825
**Vitamins**	**mg/l**
Glycine	2.00
myo-Inositol	100.00
Nicotinic acid	0.50
Pyridoxine HCl	0.50
Thiamine HCl	0.10

Establishment (pH 5.6)

DKW MEDIUM	MS3B MEDIUM	GD MEDIUM
**DKW mineral solution<br>0.5 mg/L BAP<br>100 mg/L AA<br>30g/L sucrose<br>0.1g sequestrene 138<br>8g/L Agar**	**MS3B mineral solution<br>0.5 mg/L BAP<br>100 mg/L AA<br>30g/L sucrose<br>8g/L Agar**	**GD mineral solution<br>0.5 mg/L BAP<br>100 mg/L AA<br>30g/L sucrose<br>8g/L Agar**

Multiplication (pH 5.6)

DKW MEDIUM	MS3B MEDIUM	GD MEDIUM
**DKW mineral solution<br>0.1 mg/L BAP<br>0.05 mg/L IBA<br>100 mg/L AA**	**MS3B mineral solution<br>0.1 mg/L BAP<br>0.05 mg/L IBA<br>100 mg/L AA**	**GD mineral solution<br>0.1 mg/L BAP<br>0.05 mg/L IBA<br>100 mg/L AA**

30g/L sucrose<br>0.1g sequestrene 138<br>8g/L Agar	30g/L sucrose<br>8g/L Agar	30g/L sucrose<br>8g/L Agar

<u>Elongation (pH 5.6)</u>

WPM MEDIUM

WPM mineral solution<br>0.1 mg/L ZEATIN<br>100 mg/L AA<br>30g/L sucrose<br>8g/L Agar

<u>Rooting (pH 5.6)</u>

INDUCTION ROOTING MEDIUM 1	INDUCTION ROOTING MEDIUM 2
MS1B<br>25 mg/L IBA<br>30g/L sucrose	MS1B<br>3 mg/L IBA<br>30g/L sucrose

ROOTING EXPRESSION MEDIUM

MS1B<br>30g/L sucrose<br>0.5g AC<br>8g/L Agar
